# Heritable Bovine Rumen Bacteria Are Phylogenetically Related and Correlated with the Cow’s Capacity To Harvest Energy from Its Feed

**DOI:** 10.1128/mBio.00703-17

**Published:** 2017-08-15

**Authors:** Goor Sasson, Sheerli Kruger Ben-Shabat, Eyal Seroussi, Adi Doron-Faigenboim, Naama Shterzer, Shamay Yaacoby, Margret E. Berg Miller, Bryan A. White, Eran Halperin, Itzhak Mizrahi

**Affiliations:** a Department of Life Sciences and the National Institute for Biotechnology in the Negev, Ben-Gurion University of the Negev, Beer-Sheva, Israel; b Department of Ruminant Science, Institute of Animal Sciences, Agricultural Research Organization, Volcani Center, Bet Dagan, Israel; c Department of Molecular Microbiology and Biotechnology, The George S. Wise Faculty of Life Science, Tel Aviv University, Ramat-Aviv, Israel; d Department of Animal Sciences, University of Illinois, Urbana, Illinois, USA; e The Institute for Genomic Biology, University of Illinois, Urbana, Illinois, USA; f Blavatnik School of Computer Science, Tel Aviv University, Tel Aviv, Israel; University of Hawaii at Manoa

**Keywords:** genetics, host-microbe interaction, microbial ecology, microbiome, rumen ecology

## Abstract

Ruminants sustain a long-lasting obligatory relationship with their rumen microbiome dating back 50 million years. In this unique host-microbiome relationship, the host’s ability to digest its feed is completely dependent on its coevolved microbiome. This extraordinary alliance raises questions regarding the dependent relationship between ruminants’ genetics and physiology and the rumen microbiome structure, composition, and metabolism. To elucidate this relationship, we examined the association of host genetics with the phylogenetic and functional composition of the rumen microbiome. We accomplished this by studying a population of 78 Holstein-Friesian dairy cows, using a combination of rumen microbiota data and other phenotypes from each animal with genotypic data from a subset of 47 animals. We identified 22 operational taxonomic units (OTUs) whose abundances were associated with rumen metabolic traits and host physiological traits and which showed measurable heritability. The abundance patterns of these microbes can explain high proportions of variance in rumen metabolism and many of the host physiological attributes such as its energy-harvesting efficiency. Interestingly, these OTUs shared higher phylogenetic similarity between themselves than expected by chance, suggesting occupation of a specific ecological niche within the rumen ecosystem. The findings presented here suggest that ruminant genetics and physiology are correlated with microbiome structure and that host genetics may shape the microbiome landscape by enriching for phylogenetically related taxa that may occupy a unique niche.

## IMPORTANCE

Dairy cows are an essential nutritional source for the world’s population; as such, they are extensively farmed throughout our planet and subsequently impact our environment. The microbial communities that reside in the upper digestive tract of these animals in a compartment named the rumen degrade and ferment the plant biomass that the animal ingests. Our recent efforts, as well as those of others, have shown that this microbial community’s composition and functionality are tightly linked to the cow’s capacity to harvest energy from its feed, as well as to other physiological traits. In this study, we identified microbial groups that are heritable and also linked to the cow’s production parameters. This finding could potentially allow us to apply selection programs on specific rumen microbial components that are linked to the animal’s physiology and beneficial to production. Hence, it is a steppingstone toward microbiome manipulation for increasing food availability while lowering environmental impacts such as methane emission.

## INTRODUCTION

The bovine rumen microbiome essentially enables the hosting ruminant animal to digest its feed by degrading and fermenting it. In this sense, this relationship is unique and different from the host-microbiome interactions that have evolved in humans and nonherbivorous animals, where such dependence does not exist (1, 2). This strict obligatory host-microbiome relationship, which was established approximately 50 million years ago, is thought to play a major role in host physiology (3). Despite its great importance, the impact of natural genetic variation in the host—brought about through sexual reproduction and meiotic recombination—on the complex relationship of rumen microbiome components and host physiological traits is poorly understood. Indeed, several works have reported such a link with regard to methane emission (4–7) and energy-harvesting efficiency (8–12) and, recently, associations between specific components of the rumen microbiome and animal physiology, mainly exemplified by the ability of the animal to harvest energy from its feed (13). These recent findings position the bovine rumen microbiome as the new frontier in the effort to increase the feed efficiency of dairy cows. As the human population is continually increasing, this could have important implications for food security issues as an effort toward replenishing food sources available for human consumption while lowering environmental impact on a global scale. Despite its great importance, the complex relationship of rumen microbiome components and host genetics and physiology is poorly understood. While a well-established connection exists between the structure and function of the rumen microbiome and dietary regime (14–18) as well as specific physical rumen traits such as redox potential (19, 20), until now only three studies have addressed the question of host genetics’ interaction with the rumen microbiome. In one study, PCR-denaturing gradient gel electrophoresis (DGGE) profiles of rumen microbiome samples from 18 steers of different breeds varying in their feed efficiency were compared (11). Although no direct correlation was found, some of the animals clustered as a function of their breed; therefore, it was suggested that host genetics may play a role in rumen microbiome structure. Recently, support for the interaction of host genetics with rumen microbiome composition came from a comparison of archaeon/bacterium ratios in the rumen microbiomes of eight animals from different breeds. In this study, the animals’ progeny groups were correlated with methane emissions and with archaeon/bacterium ratio (21), possibly suggesting that host genetics is connected to the ratio between these two domains. A later research study on deer hybrids showed that hybrid offspring have different microbial compositions than their parents. Additionally, alanine, arginine, proline, and phenylalanine pathways were enriched in hybrid offspring, and this enrichment was correlated with the abundance of the bacterial spp. *Prevotella*, *Acetitomaculum*, *Quinella*, *Succinivibrio*, and *Ruminobacter* (22).

Here, we explored the interaction of host genetics with bovine rumen microbiome components, with the aim of identifying specific microbes whose abundances are influenced by genetic variation in the host. We further aimed at exploring whether such identified components could be connected to rumen metabolism and host physiological attributes. To this end, we used bovine single nucleotide polymorphism chips (SNP chips) as well as bovine microbial taxa that were inferred from 16 rRNA amplicon sequencing data. We first determined the genetic relatedness between cows based on genomic SNP similarity between the animals. We then combined that information with the abundance profiles of microbiome components across animals and estimated their heritability. Our study addressed the question of whether species in the bovine rumen are heritable and the taxonomic and phylogenetic relationships of species that show measurable heritability. Finally, we asked whether the heritable microbial species may be associated with important host physiological traits and metabolic traits of the rumen.

## RESULTS

### Experimental design and data.

Our main goal was to identify microbial species where significant proportions of their variation in abundance profiles can be attributed to heritable genetic factors. To achieve this, we analyzed common SNP genotyping information of 47 Holstein-Friesian dairy cows. We consolidated this information with additional data for these animals from our recent study (13) in which we have discovered microbiome mechanisms that underlie energy-harvesting efficiency from feed in bovines. For each animal, we sequenced the 16S rRNA gene from rumen samples from three consecutive days. We also quantified rumen metabolites and performed rumen metabolic activity assays such as *ex vivo* rumen methane production and fiber digestion measurements. We also consolidated metadata of individual cows’ production indices and physiological indices. Low-quality and noninformative SNPs were removed using a quality control (QC) pipeline (see Materials and Methods). 16S amplicon sequencing analysis was performed using the QIIME pipeline (23). The rumen microbiome taxonomic profiles represented by 85,255 species-level operational taxonomic units (OTUs) (three samples per animal) were associated with genomic data represented by genotyping of common SNP loci (see Materials and Methods). Notably, we focused on identification of heritable microbial OTUs rather than the heritability estimate magnitude. This approach is more robust to heritability estimate values that are typical with small sample sizes in estimation procedures. The microbial OTUs found to be associated with the animals’ genomes were further correlated with metabolomics data of the microbiomes, as well as with animal physiology and productivity parameters.

### Heritable species have high phylogenetic relatedness and are enriched with the order *Bacteroidales.*

**FIG 1:**
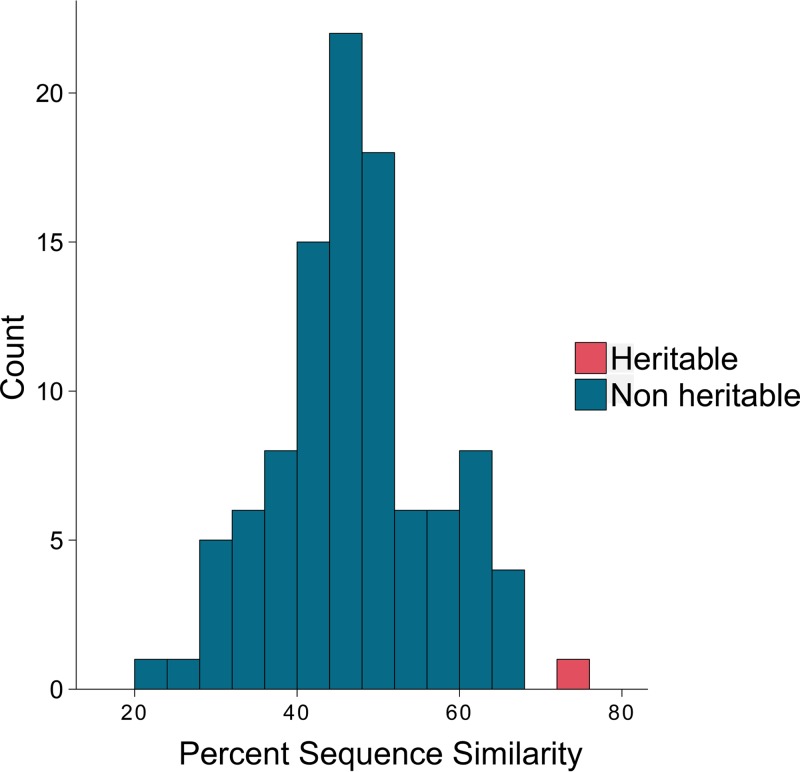
The heritable portion of the bovine rumen microbiome is phylogenetically closely related. The mean pairwise similarity in the 16S rRNA gene sequence of randomly selected groups of rumen OTUs of the same size (*n* = 22) was compared to the mean pairwise similarity of the 22 heritable OTUs. The *y* axis represents the number of groups, and the *x* axis represents the sequence similarity. The group of heritable OTUs with a calculated mean similarity of 72% at the 16S rRNA gene sequence is depicted in pink. The distribution of randomly selected groups of rumen OTUs is depicted in blue. All random groups showed lower mean 16S rRNA gene similarity (*P* < 0.01).

The first step in our analysis was to identify heritable bacterial species; that is, in our context, to identify microbial species where significant proportions of their variation in abundance profiles could be attributed to heritable genetic factors. This would be reflected in a highly similar abundance of certain species among animals that share a similar genetic background. Accordingly, we estimated the relatedness between all pairs of animals in the cohort. This estimation was done by considering both the count and the infrequency of the alleles (SNPs) in the reference genotypes. We used these pairwise genetic relationship estimates together with each species’ abundance profile to calculate their heritability estimate (Fig. 1).

To increase the confidence of our analysis, we limited our heritability analysis exclusively to OTUs which were present in at least 12 genotyped animals (25% of the genotyped subset) as previously described (24). In addition, we performed three independent heritability analyses for each OTU, one for each sampling day. Only OTUs that exhibited a significant heritable component (heritability estimate of >0.7 and *P* value of <0.05) in all three individual sampling days were considered heritable OTUs. Following this procedure, our analysis resulted in 22 heritable OTUs that match these criteria (see Fig. S3 in the supplemental material), all belonging to the bacterial domain. Although the heritability significance assessment procedure is based on a parametric test, we inspected the robustness of this finding by examining the false discovery rate distribution of the test under permuted assumptions. For that purpose, we generated a null model with 100 iterations, where in each iteration we repeated the heritability analyses after randomly shuffling the genetic profile order. In 94% of the permutations, the number of OTUs detected as heritable was smaller than 22, while in most permutations, the number of OTUs detected as heritable was under 5 (Fig. S1).

10.1128/mBio.00703-17.1 FIG S1  Histogram showing the false discovery rate of the heritability test under permuted assumptions, based on 100 random permutations. *x* axis, number of heritable OTUs detected; *y* axis, number of permutations. A red vertical line presents the number of OTUs detected as heritable in the actual (nonpermuted) data. Download FIG S1, PDF file, 0.1 MB. Copyright © 2017 Sasson et al. 2017 Sasson et al. This content is distributed under the terms of the Creative Commons Attribution 4.0 International license.

10.1128/mBio.00703-17.2 FIG S2  A null model for testing the significance of the actual Spearman correlation *r* values of the heritable OTUs with each of the indices. Red bars represent values resulting from the actual heritable OTUs’ relative abundance profile; black bars represent the values derived from 1,000 permutations. In each such permutation, each OTU’s abundance profile was randomly shuffled. Download FIG S2, PDF file, 0.1 MB. Copyright © 2017 Sasson et al. 2017 Sasson et al. This content is distributed under the terms of the Creative Commons Attribution 4.0 International license.

10.1128/mBio.00703-17.3 FIG S3  Fifty OTUs with top heritability estimates ordered by number of days (the microbiome was sampled on three different days) in which the heritability estimate was found significant as the primary sorting key and the mean heritability estimate was found significant as the secondary sorting key. *x* axis, sampling day; *y* axis, microbial OTU taxonomy. The red line signifies the threshold above which OTUs were considered heritable. Download FIG S3, PDF file, 0.1 MB. Copyright © 2017 Sasson et al. 2017 Sasson et al. This content is distributed under the terms of the Creative Commons Attribution 4.0 International license.

**FIG 2:**
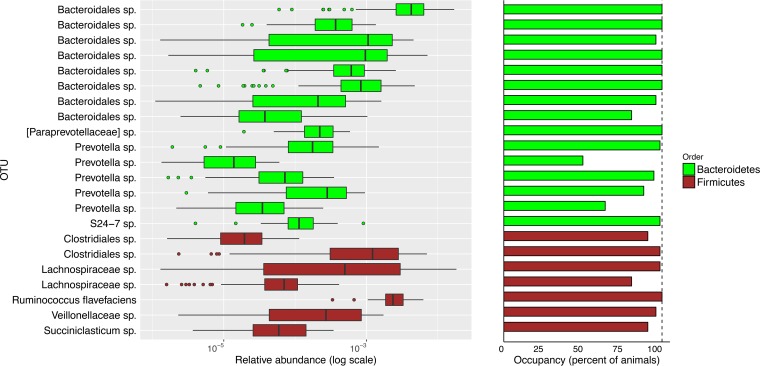
Heritable OTUs show a high presence. OTUs with their taxonomy annotations are listed on the left. The relative abundance of each OTU along the cohort of cows is presented in the left panel, and the presence of each OTU is displayed in the right panel. Green indicates an OTU from the *Bacteroidales* order, while brown indicates an OTU from the *Clostridiales* order.

It is interesting that the heritable OTUs exhibited a high presence across animals, ranging between 50% and 100% of the animals, with the majority appearing in 70 to 100% of the examined animals (Fig. 2; Fig. S4 and S5). The abundance profile of the heritable microbes was correlated with their presence profile (Spearman correlation between the presence counts and abundance sums, *r* = 0.75, *P* < 5 × 10^−5^).

10.1128/mBio.00703-17.4 FIG S4  Heat map depicting the abundance profiles of heritable microbial OTUs (mean from three sampling days) along the entire study cohort. *x* axis, animal identification number in the experiment; *y* axis, taxonomy of microbial OTUs. The heat map is colored according to the relative abundance of the given OTU in the given animal. Relative abundance is multiplied by 1,000,000 to ease readability. The red horizontal line signifies the threshold above which OTUs were considered heritable. Download FIG S4, PDF file, 0.1 MB. Copyright © 2017 Sasson et al. 2017 Sasson et al. This content is distributed under the terms of the Creative Commons Attribution 4.0 International license.

10.1128/mBio.00703-17.5 FIG S5  Heat map depicting the abundance profiles of heritable microbial OTUs (mean from three sampling days) along the genotyped subset of the study cohort. *x* axis, animal identification number in the experiment; *y* axis, microbial OTU taxonomy. The heat map is colored according to the relative abundance of the given OTU in the given animal. Relative abundance is multiplied by 1,000,000 to ease readability. The red horizontal line signifies the threshold above which OTUs were considered heritable. Download FIG S5, PDF file, 0.1 MB. Copyright © 2017 Sasson et al. 2017 Sasson et al. This content is distributed under the terms of the Creative Commons Attribution 4.0 International license.

When we measured the phylogenetic distance between these OTUs, we found that they were highly phylogenetically related on the basis of the similarity of their 16S nucleotide sequences (Fig. 1).

These OTUs belong to the two main phyla of the rumen microbiome, namely, *Bacteroidetes* and *Firmicutes*, and grouped under the two dominant orders in the rumen, *Bacteroidales* and *Clostridiales* (Fig. 2).

We further asked whether this phylogenetic composition of heritable OTUs represents that of the overall species composition in the rumen. Here, we found that the order *Bacteroidales* is represented by more species within the heritable OTUs than in the overall rumen microbiome (trend, Fisher exact test, *P* < 0.053).

### Heritable bacterial abundance is correlated with host traits as well as with rumen metabolic parameters and can significantly explain a high proportion of the phenotypic variation between animals.

**FIG 3:**
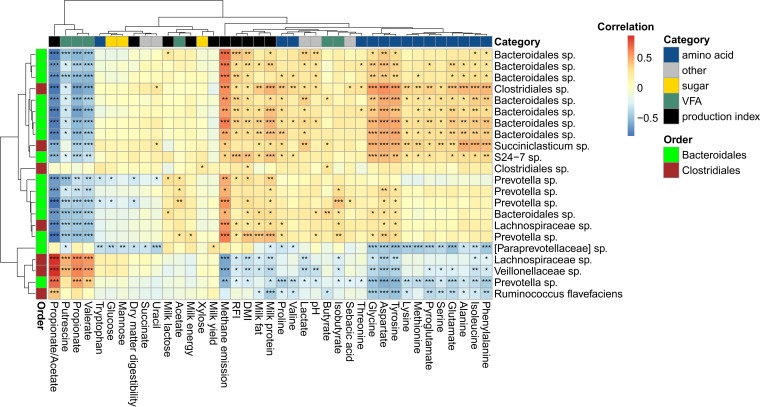
Heritable OTUs are correlated with host attributes and rumen metabolites. A heat map describing the Spearman correlation between the relative abundance of rumen heritable OTUs (rows) and selected indices representing different physiological attributes of the host or rumen metabolites (columns). OTUs are color coded by order (green represents *Bacteroidales*, and brown represents *Clostridiales*). Physiological attributes are colored in black, and rumen metabolites are color coded according to four groups: amino acids (blue), sugars (yellow), VFAs (green), and all other measured metabolites (gray). *, **, and *** represent nominal *P* values smaller than 0.05, 0.005, and 0.0005, respectively.

Following our recent work in which we found microbiome components that are connected to energy-harvesting efficiency and other physiological parameters of the host (13), we hypothesized that heritable taxa that are correlated with the host genome will potentially be related to rumen metabolism as well as to host physiology. Hence, we looked for a correlation between heritable microbes and all measured physiological parameters of the animals, as well as with rumen metabolic parameters. In detail, we correlated the abundance profile along the cohort of 78 cows of each heritable OTU with the profile of each measured index (a rumen metabolite or other index). We then compared the mean correlation of heritable OTUs with each of the rumen metabolites and host physiological attributes to a null model. In each of 1,000 iterations of the null model, we shuffled each heritable OTU’s abundance profile and recalculated its mean correlation with each of the rumen metabolites and host physiological attributes. This analysis revealed that the heritable OTUs exhibit a strong and significant correlation with many of the rumen metabolic parameters, as well with physiological attributes of the host (Fig. 3; Fig. S2).

**FIG 4:**
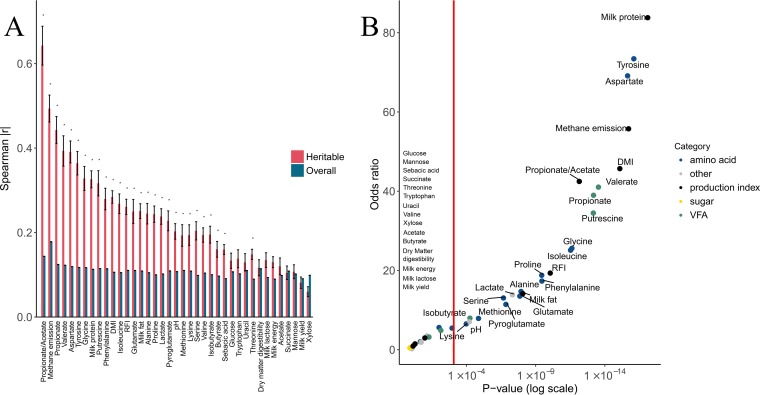
Heritable OTUs are more closely connected to host physiology and rumen metabolites than other rumen microbes. (A) The mean absolute correlation (Spearman) of the heritable OTUs with a given index is compared with that of the entire microbiome. Asterisks represent significant differences in means (*t* test, *P* < 0.05). Red bars represent correlations of the heritable microbiome, while the blue bars represent correlations of the entire microbiome. (B) The odds ratio for an OTU to be correlated with a given index (nominal Spearman *P* of <0.05), between the heritable OTUs and all OTUs. *y* axis, odds ratio; *x* axis, *P* value derived from Fisher’s exact test. The red vertical line defines the Bonferroni-corrected 0.05 significance threshold. Point colors signify category according to the legend.

With relation to rumen metabolism, the strongest correlations for the heritable OTUs were with propionate/acetate ratio (highest-magnitude *r* = 0.86, mean |*r*| = 0.64), methane metabolism (highest-magnitude *r* = 0.69, mean |*r*| = 0.49), propionic acid (highest-magnitude *r* = −0.6245274, mean |*r*| = 0.44), and valeric acid (highest-magnitude *r* = −0.57, mean |*r*| = 0.39), as well as with the concentration of several amino acids, namely, glycine, aspartate, and tyrosine (with highest-magnitude *r* = 0.51, 0.5, and −0.53 and mean |*r*| = 0.32, 0.39, and 0.36, respectively). Concerning host attributes, the best-correlated parameters were the milk protein (highest-magnitude *r* = 0.46, mean |*r*| = 0.33), dry matter intake (DMI) (highest-magnitude *r* = 0.41, mean |*r*| = 0.28), feed efficiency (represented by residual feed intake [RFI], highest-magnitude *r* = 0.26, mean |*r*| = 0.39), and milk fat (highest-magnitude *r* = 0.39, mean |*r*| = 0.25). Moreover, when we inspected the individual correlation of the heritable OTUs with propionate/acetate ratio, methane metabolism, propionic acid, and valeric acid, the majority of these OTUs were correlated either positively or negatively with these parameters (Fig. 4A). Regarding host physiological attributes, the majority of heritable OTUs were positively correlated with RFI, DMI, and milk protein.

These findings raised the question of whether the portion of heritable microbes that are correlated with host physiology and rumen metabolism is different from the one found in the overall rumen microbiome, as in our recent study we found that the rumen microbiome is tightly linked to many of the host attributes and rumen metabolism parameters (13). To this end, we calculated the OTU correlation odds ratio for each index (see Materials and Methods); in this analysis, we identified significantly higher odds for an OTU to be correlated with a given index within the heritable microbiome for many parameters. This was especially true for the parameters with which these heritable microbes showed high correlation (Fig. 4B).

**FIG 5:**
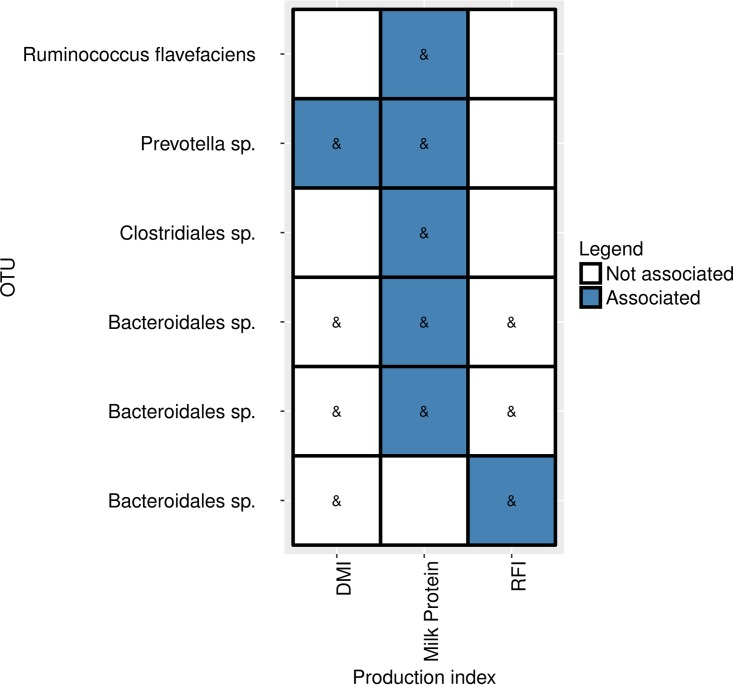
A portion of heritable OTUs were found to be associated with host physiology in a previous study (13). Six of the 22 heritable OTUs that were associated in our previous study with different cow production indices, namely, dry matter intake (DMI), milk protein, and feed efficiency, measured as residual feed intake (RFI). OTUs and their taxonomy are in the rows, and the production indices are in the columns. The ampersand inside a tile indicates that a significant correlation was found, in the current study, between the heritable OTU and the production index.

One of the heritable OTUs with the phylogenetic association of *Bacteroidales*, which was found to be heritable and highly correlated with the feed efficiency trait in this study, was independently found to be correlated with this trait in our previous study (13) (Fig. 5). Additionally, five other heritable OTUs with the phylogenetic associations of *Bacteroidales*, *Prevotella*, *Clostridiales*, and *Flavefaciens* were found to be highly correlated with milk protein in our previous study, and one OTU of the genus *Prevotella* mentioned above was also found to be significantly correlated with dry matter intake (DMI) in our previous study (Fig. 5).

### Rumen and animal physiology traits show various heritability estimates.

After identifying heritable microbial species that exhibit correlation with host traits, we were interested in estimating the heritability of the different important host and rumen metabolism traits with which we found the heritable microbes to be correlated (Fig. S6). The volatile fatty acids (VFAs) propionate, succinate, and valerate along with milk protein with the efficiency measures of RFI and DMI exhibited significant heritability estimates. None of the other traits exhibited significant heritability, suggesting that if the cohort size were increased, this trait might prove to be significantly heritable. Alternatively, the current small sample size in our study could also explain the higher estimates for RFI (25) and milk protein (26) than previously published estimates due to intrinsic confounders with the current way of analysis.

10.1128/mBio.00703-17.6 FIG S6  Heritability estimates of host traits. *x* axis, traits; *y* axis, heritability estimate. A star on the top of the bar signifies significant heritability estimate. Download FIG S6, PDF file, 0.1 MB. Copyright © 2017 Sasson et al. 2017 Sasson et al. This content is distributed under the terms of the Creative Commons Attribution 4.0 International license.

## DISCUSSION

Our study underlines that the bovine rumen microbiome includes heritable components. We increased resolution over previous studies by applying SNP-based heritability estimates, combined with amplicon sequencing data, host traits, and rumen metabolites. Although we set our threshold for examining microbes that exist in at least 12 genotyped animals (16% of the study cohort), the microbes that were found to be heritable exhibited a considerably higher presence, ranging between 50 and 100% of the animals. This finding may suggest that these microbes are extremely important to rumen metabolism and therefore also to host physiology. These relationships to the host’s metabolism and physiology may explain the potential association of these microbial species with the host’s genome. However, it should still be noted that in humans and mouse models, microbial taxa showing high narrow sense heritability (*h*^2^) do not necessarily lead to significant associations by quantitative trait locus (QTL) analysis or genome-wide association study (GWAS) (27). Whether this is due to simply missing heritability or to false discovery remains to be determined.

Our results show that the heritable microbial species represent a related phylogenetic group (Fig. 1). This finding corresponds with a fundamental ecological notion that organisms that share a similar ecological niche are more prone to be phylogenetically close to each other than organisms not sharing the same niche (28). Hence, it is tempting to speculate that the apparent relatedness of the heritable microbial species suggests that they occupy similar ecological niches within the host and potentially share mechanisms of interaction with it, which are affiliated with their phylogenetic association (Fig. 2).

We observed that the metabolites and physiological parameters measured are generally clustered together according to their category, based on hierarchical clustering of their correlation profile for the different heritable OTUs (columns in the heat map [Fig. 3]). For example, most amino acids cluster together, some volatile fatty acids pair together, and six out of nine production indices neighbor each other along the heat map. At the same time, from the heritable OTU perspective, even within the distinct niche of heritable OTUs identified in this study, one can see that the clustering of OTUs according to their abundance profiles (rows in the heat map, Fig. 3) separates them to a high degree according to their taxonomic affiliations, e.g., eight of nine unknown *Bacteroidales* OTUs cluster together and three of five *Prevotella* OTUs cluster together. This finding corresponds with our previous study where we show that specific microbial lineages are correlated with specific physiological traits of the host (8).

Some of the heritable bacteria that were found to be correlated with specific host traits, notably DMI, energy-harvesting efficiency (RFI), and milk protein, were also found independently in our recent study to be connected to these traits, further strengthening these findings (Fig. 5). Intriguingly, we found that the heritable bacteria contain higher proportions of microbes correlated with host traits and with rumen metabolic parameters (Fig. 4A and B). These findings suggest that host genetic variation can have a measurable impact on physiological traits of the host as well as on rumen metabolism by potentially modulating the abundances of different groups of rumen microbes. These findings indicate that host genetics are associated with specific rumen bacteria, which are potentially more prone to influence rumen metabolism and host physiology. Notably, the metabolites and host traits that were found to be correlated with heritable bacteria were also connected by their metabolism. This could be seen in the correlation values of methane production, propionate/acetate ratio, lactate, propionate, and butyrate, as well as energy-harvesting efficiency of the host (represented as RFI), which are correlated with the heritable bacteria. These metabolites were previously shown to be connected to each other by their metabolic pathways (29–31), and the balance between them could affect energy-harvesting efficiency, as was shown in our recent study (13). It is specifically interesting to see that the heritable bacteria are mostly correlated with the propionate/acetate ratio (mean |*r*| = 0.64), which is inversely correlated with methanogenesis and lactate while being positively correlated with RFI, which estimates energy-harvesting efficiency (Fig. 3 and 4). These findings add the host genotype as another dimension to our published findings (13).

In that study, we showed that the metabolism of lactate into VFAs is connected to methane production and increase in energy-harvesting efficiency. These findings were also recently supported by an independent study (32). Another noteworthy finding is the milk protein trait which was correlated with heritable microbes and exhibited the highest odds ratio, pointing to enrichment of heritable bacteria connected to this host trait (Fig. 4). This connection could potentially be explained by the several amino acid rumen concentrations that were also linked to the heritable bacteria, as it was shown elsewhere that increases in amino acids that are not anabolized in the rumen are connected to increases in milk protein (33). Our observations further strengthen the notion of a triangular relationship among the host genotype, rumen bacteria, and host traits. Although it is tempting to speculate that host genetics mediate control of physiological attributes via rumen metabolism, the relationship between these parameters is still to be determined. To target these concerns, future experiments with a larger sample size should be performed to obtain more accurate heritability estimations for both microbes and host traits. Such accuracies could then be utilized to compare heritability estimates of microbes and host traits and suggest cases of the direct causal roles in cases of equal scores. A more direct approach to tackle the question of causality would be to apply transplantation of rumen microbes in different host genetic backgrounds.

Our study presented here sheds light on yet-unanswered questions regarding host microbiome interactions and highlights that host genetic variation is associated with specific microbes. It presents an intricate, unsolved relationship between host genetics, specific microbes, rumen metabolism, and host attributes that will be deciphered by future research involving larger animal cohorts and experimental setups that will allow distinguishing of cause and effect.

## MATERIALS AND METHODS

### Microbial DNA extraction.

The microbial fraction of the rumen fluid was separated according to the method of Stevenson and Weimer (34), with the minor modifications that were performed by Jami et al. (35). DNA was extracted as described by Stevenson and Weimer (34).

### Genomic DNA extraction.

Five hundred microliters of whole blood from each individual animal was mixed with 500 μl Tris-HCl-saturated phenol (pH 8.0) and 500 μl of double-distilled water (DDW). The mixture was shaken for 4 h at room temperature and subsequently centrifuged at 7,500 × *g* for 5 min, and the aqueous phase was transferred to a new tube with 500 μl of Tris-HCl (pH 8.0)-saturated phenol-chloroform (1:1) and subsequently centrifuged at 7,500 × *g* for 5 min. The aqueous phase containing the DNA was transferred to a new tube for further processing.

### Animal genotyping.

The animals are members of the Volcani Center herd of the Agricultural Research Organization, Israel. Within the genotyped animals, there were 11 groups each sharing a common sire (groups of half-siblings). One such group consisted of four half-siblings, another consisted of three half-siblings, and all the rest of the the groups consisted of two half-siblings. Additionally, there were two pairs of half-siblings sharing a common dam. There were no full siblings among the genotyped animals. As a DNA microsatellite-assisted survey of incorrect paternity attribution within the Israeli dairy cattle population revealed that such incorrect attributions are not rare (36), we opted to base our estimation of genetic relatedness between the cows solely on their genomic information.

Genomic DNA extracts from the animals were loaded into a bovine SNP 50K chip, which is targeted at 54,609 common SNPs that are evenly spaced along the bovine genome (Illumina). The SNP chip model used was Illumina bovine SNP50-24 v3.0, catalog no. 20000766, and it was processed according to the manufacturer’s protocol (37) at the Genomics Center of the Biomedical Core Facility, Technion, Israel.

### 16S rRNA gene sequencing and analysis.

Amplification of the 16S V2 region was performed with primers CCTACGGGAGGCAGCAG (forward) and CCGTCAATTCMTTTRAGT (reverse). The libraries were then pooled and subsequently sequenced on a single MiSeq flow cell (Illumina) for 251 cycles from each end of the fragments, following analysis with Casava 1.8. A total of 49,760,478 paired-end reads were obtained from the total sample, with an average of 106,325 paired-end reads per sample. The QIIME (23) pipeline version 1.7.037 was used for data quality control and analyses. OTU analysis was performed on species clusters (97% identity) that were created using UCLUST (38). OTUs were subjected to taxonomy assignment using BLAST (39) against the 16S rRNA reference database RDP (40). Singletons and doubletons were filtered from the data set, resulting in 85,255 species with an average of 5,039 per sample.

### Genotype data quality control.

Genotypes of 47 individuals from the current analysis were combined with a reference set of 2,691 individual genotypes that were collected from individual Holstein-Friesian dairy cows in farms all over Israel and the Netherlands (courtesy of the Israeli Cow Breeders Association [ICBA]). The reference set of genotypes allowed for more robust quality control (QC) and for the creation of the generic relationship matrix. QC was performed with the PLINK (41) program, with the following parameters: *-cow—file isgenotype_all—maf 0.05—geno 0.05—mind 0.05 –out isgenotype_all_qc—recode12.* SNPs that were not genotyped in more than 5% of the individuals were removed. Similarly, individuals were removed from the analysis if they had been genotyped in less than 95% of the loci (SNPs) covered by the SNP chip.

Three hundred fifty-four individuals (1 belonging to the study group) were removed because of low genotyping, 3,797 SNPs were removed because of “missingness” in the genotyped populations, and 11,290 SNPs failed the minor allele frequency (MAF) criteria. The total number of SNPs passing QC was 40,812.

### Generation of genetic relatedness matrix.

All animals and SNPs that passed QC were used to generate a matrix that estimates the genetic relatedness between each unique pair of animals. The GCTA (42) software was used to calculate the relationship matrix. The matrix is based on the count of shared alleles, weighted by the allele’s rareness:

$$A_{jk}=\frac{1}{n}\overset{n}{\underset{i\;=\;1}{\sum }}\left(\frac{\left(x_{ij}-2p_{i})(x_{ik}-2p_{i}\right)}{2p_{i}(1-p_{i})}\right)$$

where *A*_*jk*_ represents the genetic relationship estimate between animals *j* and *k*; *x*_*ij*_ and *x*_*ik*_ are the counts of the reference alleles in animals *j* and *k*, respectively; *p*_*i*_ is the proportion of the reference allele in the population; and *n* is the total number of SNPs used for the relatedness estimation.

### Heritability estimates.

Heritability estimates of each species were established upon the distribution of the relative abundance of the species in question in conjunction with the estimated genetic relatedness between the animals. The estimation was performed using the software GCTA (42, 43). The model used by this software is termed total heritability and reflects the heritability explained by all the SNPs that passed QC. The model is *y* = *X*β + *W*u + ε, where *y* is the vector of observations (phenotypes), β is a vector of fixed effects (study covariates), *X* is the design matrix, u is the vector of SNP effect, *W* is the standardized genotype matrix, and ε is the individual (residual) effect.

Then, the variance in the model could be attributed to two sources, genetic and random error, in the following manner:

$$V=WW\prime \sigma_{u}^{2}+I\sigma_{\varepsilon }^{2}$$

where *V* is the overall variance, *I* is the identity matrix (*n* × *n*), $\sigma_{u}^{2}$ is the variance due to genetics (overall SNP effects), and $\sigma_{\varepsilon }^{2}$ is the variance due to individual effects (residual). Next, GCTA estimates the value of $\sigma_{u}^{2}$ and $\sigma_{g}^{2}$, and the heritability is then estimated as

$$\begin{array}{l} h^{2}=\frac{\sigma_{u}^{2}}{\sigma_{u}^{2}+\sigma_{\varepsilon }^{2}} \end{array}$$

### Comparing phylogenetic distance within heritable bacterial OTUs to that within overall rumen microbiome.

DNA similarity (percent) between each unique pair within the 22 heritable bacterial OTUs was calculated using Clustal W v2 (44), and the mean of these similarities was then calculated. A reference range of mean similarities was calculated by randomly sampling 100 subsets of the same size, each (*n* = 22) drawn from the pool of OTUs appearing in at least 12 genotyped animals (9,282). Pairwise DNA similarities and their means were calculated for each random subset. To draw significance for the mean similarity within the group of heritable bacterial OTUs, we ranked its mean similarity within all 100 mean similarity values that were obtained from the random subsets.

### OTU correlation odds ratio.

The OTU correlation odds ratio is (hc/hn)/(nc/nn), where hc is the count of heritable OTUs correlated with the index, hn is the count of heritable OTUs not correlated with the index, nc is the count of nonheritable OTUs correlated with the index, and nn is the count of nonheritable OTUs not correlated with the index. In this context, OTU was correlated with the index if it had a nominal Spearman *P* value of <0.05.

### Statistics and plots.

Statistical analysis was performed using R (45) software, and plots were produced using the ggplot2 (46) and pheatmap (47) packages.

10.1128/mBio.00703-17.7 TABLE S1  OTU table of 50 OTUs with highest heritability estimates along the entire study cohort. Download TABLE S1, TXT file, 0.1 MB. Copyright © 2017 Sasson et al. 2017 Sasson et al. This content is distributed under the terms of the Creative Commons Attribution 4.0 International license.

10.1128/mBio.00703-17.8 TABLE S2  OTU table of 50 OTUs with highest heritability estimates along the genotyped subset. Download TABLE S2, TXT file, 0.1 MB. Copyright © 2017 Sasson et al. 2017 Sasson et al. This content is distributed under the terms of the Creative Commons Attribution 4.0 International license.
