# Supplementary figures and images for: Heritable Bovine Rumen Bacteria Are Phylogenetically Related and Correlated with the Cow’s Capacity To Harvest Energy from Its Feed
Source: mBio. 2017 Aug 15;8(4):e00703-17. doi: 10.1128/mBio.00703-17 (PMC5559629; doi:10.1128/mBio.00703-17)

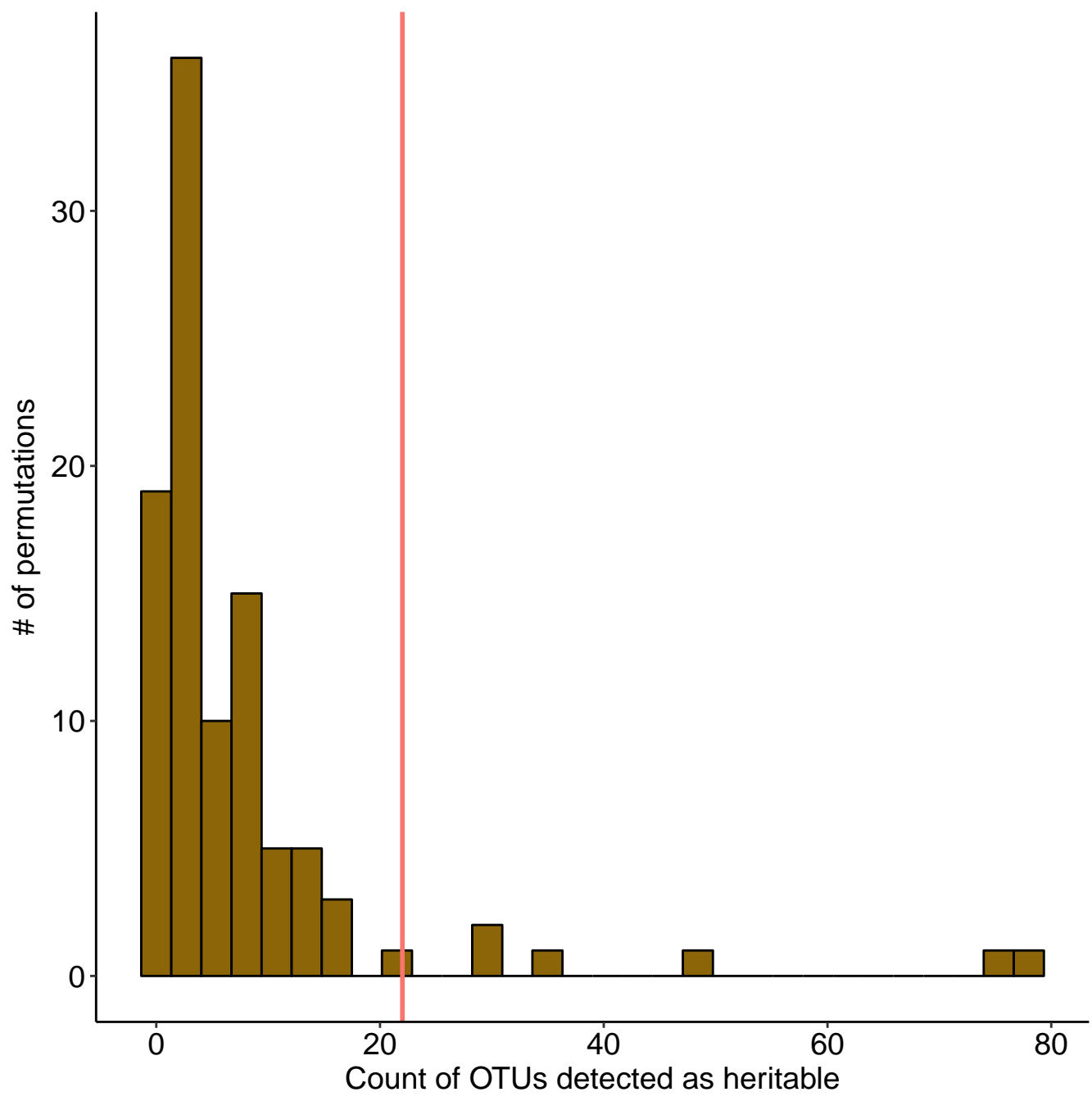

Supplement: FIG S1 [file mbo004173383sf1.pdf]

OTU Taxonomy

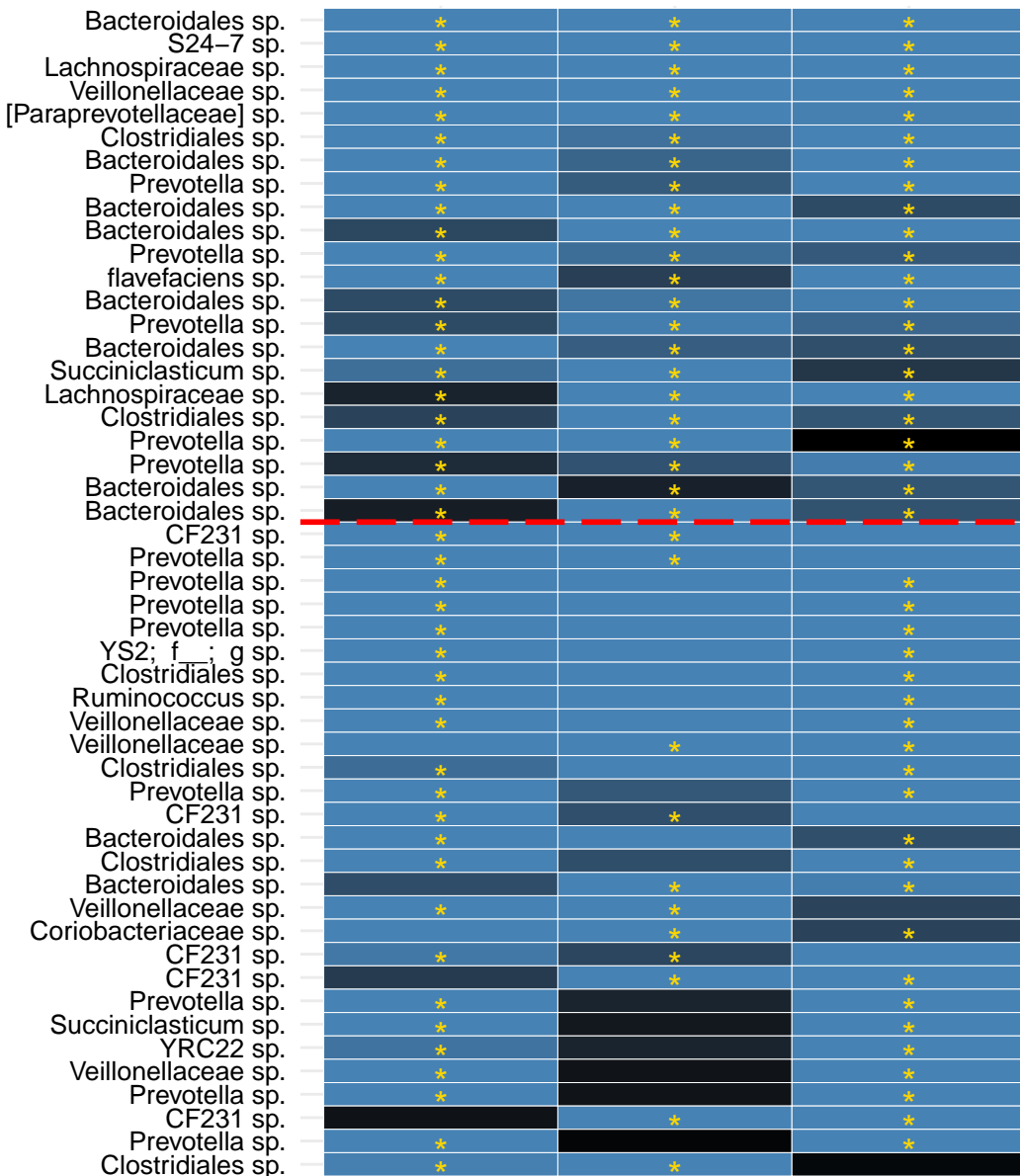Heritability  
estimate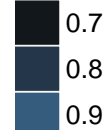

Sampling date

Supplement: FIG S3 [file mbo004173383sf3.pdf]

OTU Taxonomy

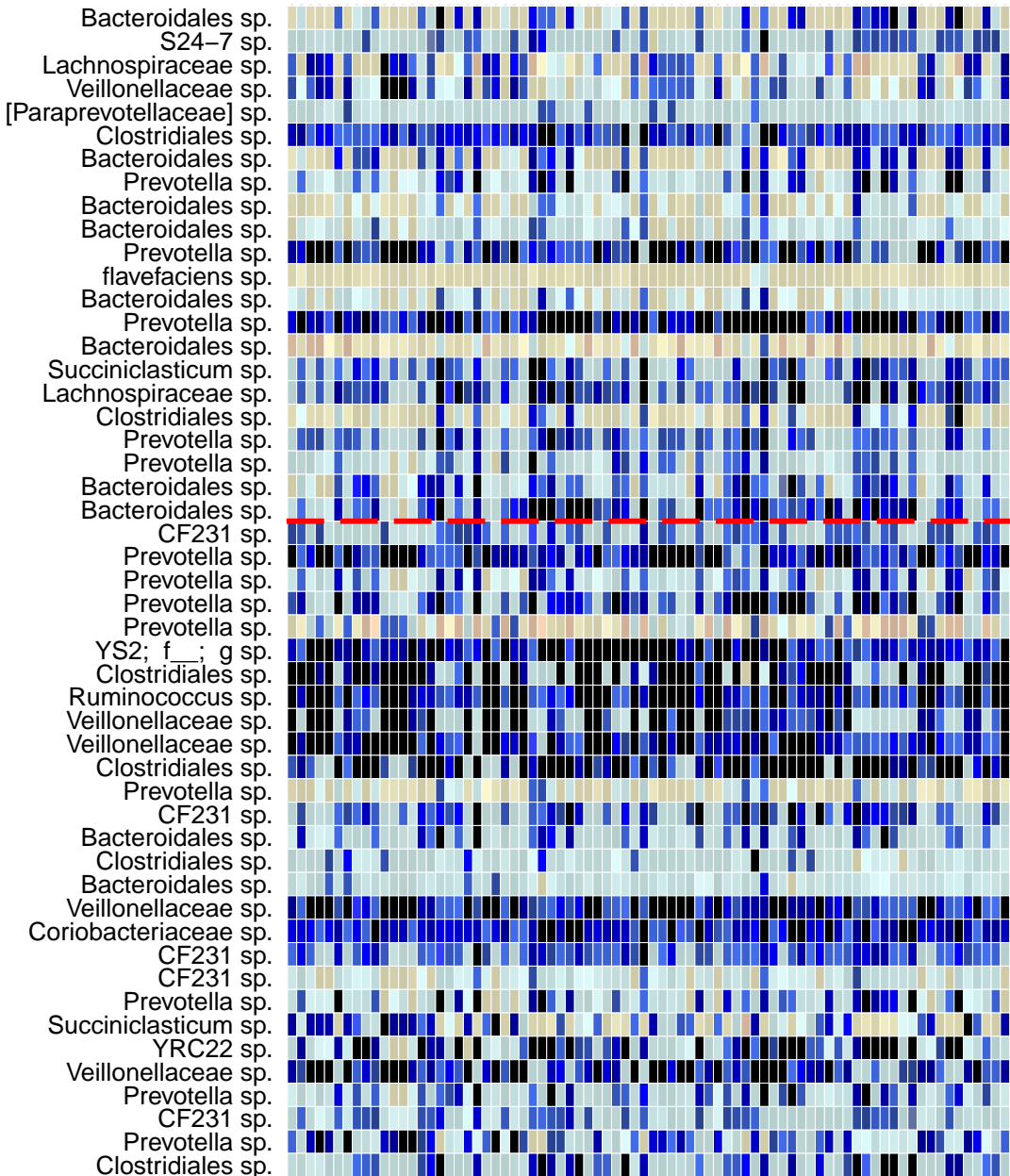

Cow ID

Supplement: FIG S4 [file mbo004173383sf4.pdf]

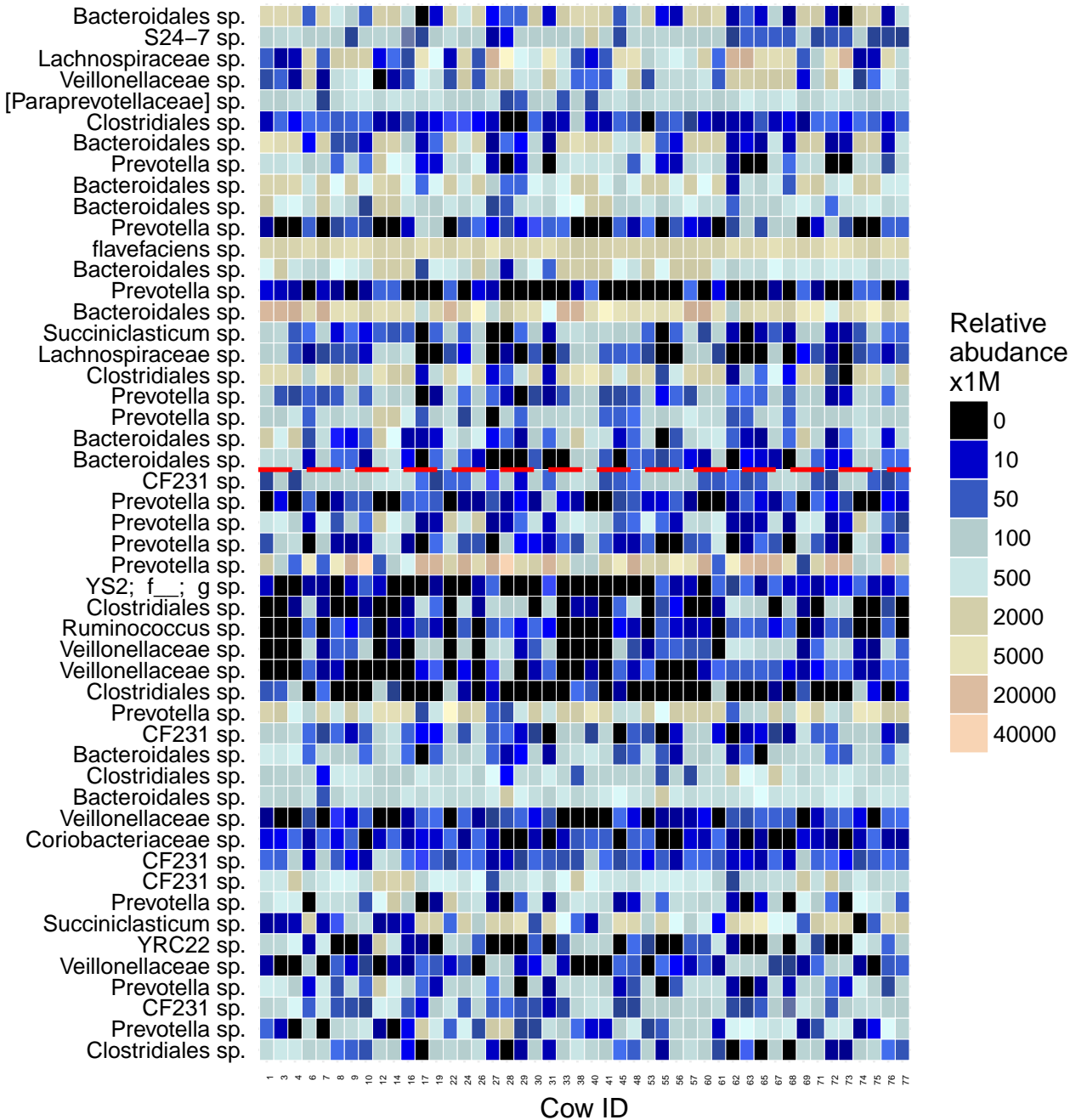

Supplement: FIG S5 [file mbo004173383sf5.pdf]

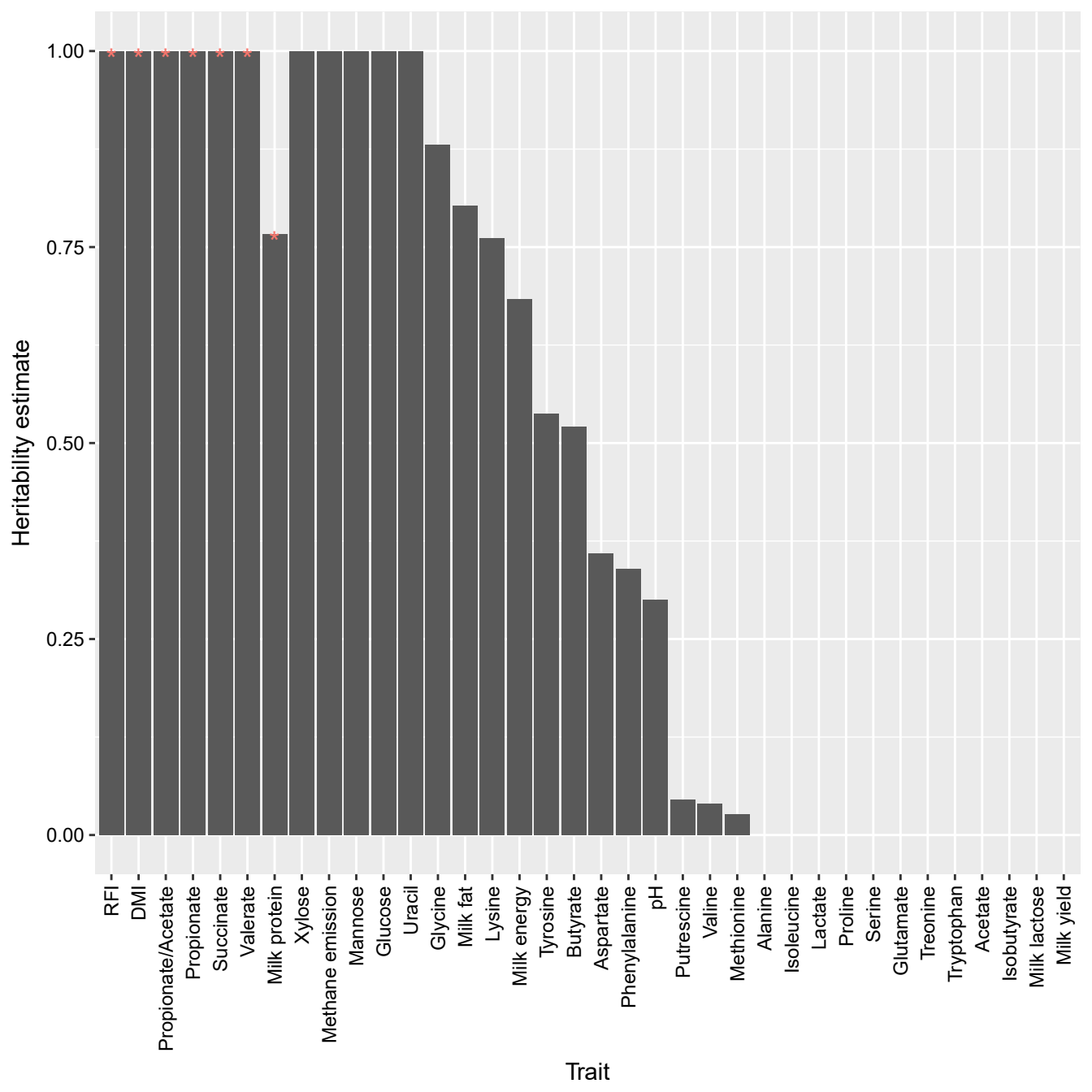

Supplement: FIG S6 [file mbo004173383sf6.pdf]
