# Supplementary material for: Heritable Bovine Rumen Bacteria Are Phylogenetically Related and Correlated with the Cow’s Capacity To Harvest Energy from Its Feed
Source: mBio. 2017 Aug 15;8(4):e00703-17. doi: 10.1128/mBio.00703-17 (PMC5559629; doi:10.1128/mBio.00703-17)
Supplement: FIG S2 [file mbo004173383sf2.pdf]

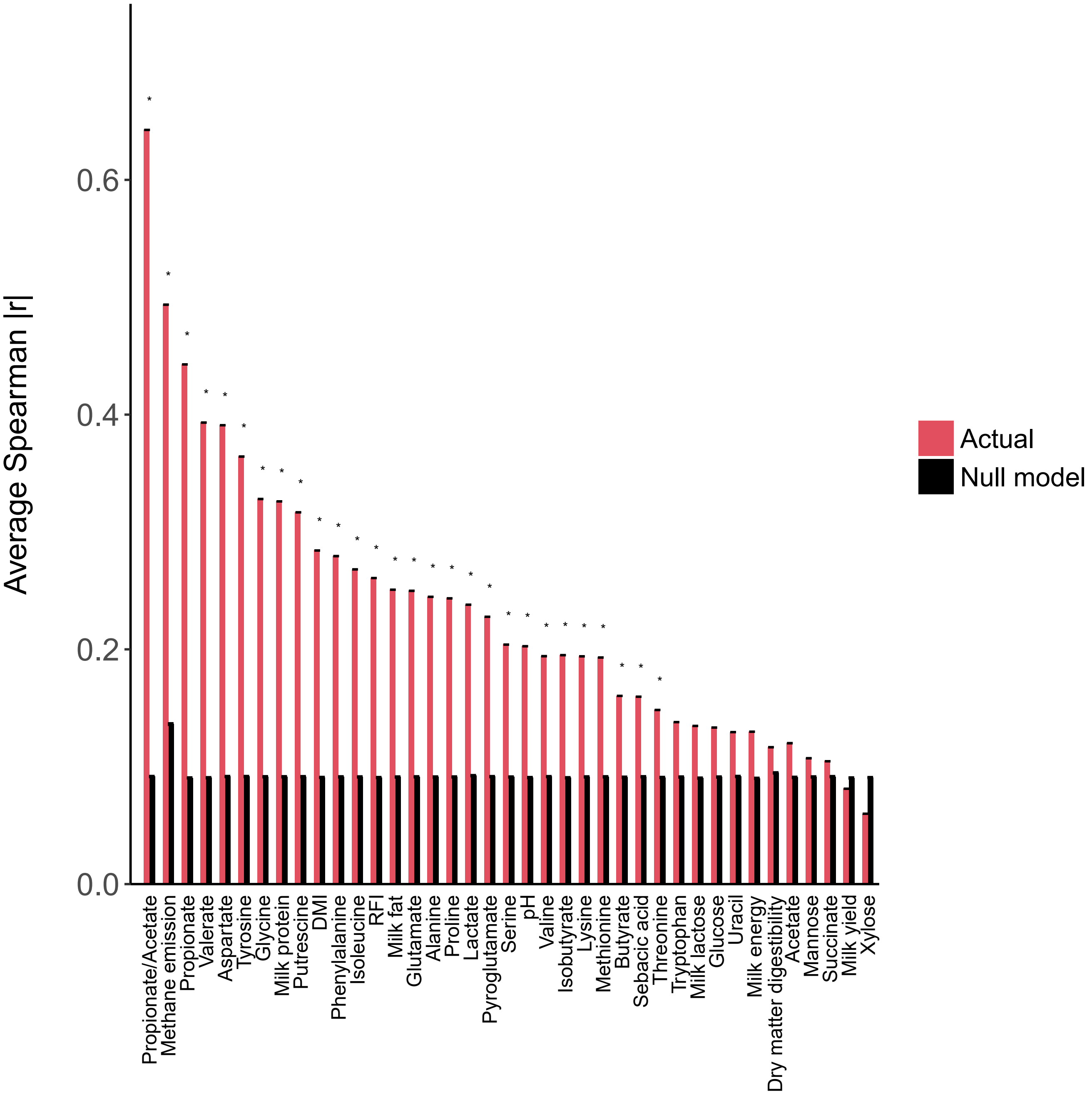
Average Spearman |r|
0.0
0.2
0.4
0.6
Actual
Null model
Propionate/Acetate
Methane emission
Propionate
Valerate
Aspartate
Tyrosine
Glycine
Milk protein
Putrescine
DMI
Phenylalanine
Isoleucine
RFI
Milk fat
Glutamate
Alanine
Proline
Lactate
Pyroglutamate
Serine
pH
Valine
Isobutyrate
Lysine
Methionine
Butyrate
Sebacic acid
Threonine
Tryptophan
Milk lactose
Glucose
Uracil
Milk energy
Dry matter digestibility
Acetate
Mannose
Succinate
Milk yield
Xylose
